# Changing patterns of respiratory pathogens in hospitalized children with community-acquired pneumonia in northern China following the lifting of non-pharmaceutical interventions

**DOI:** 10.1128/spectrum.01279-25

**Published:** 2025-09-24

**Authors:** Ting-ting Jiang, Ze-ming Wang, He Tang, Lin Sun, Qian Han, Hui Qi, Tian-yi Wang, Jing Xiao, Chenxi Li, Xue-mei Yang, Sai Zhao, Xue Tian, Hui Wang, Xu Li, Jing Bi, Wei-wei Jiao, A-dong Shen

**Affiliations:** 1 Baoding Key Laboratory for Precision Diagnosis and Treatment of Infectious Diseases in Children, Hebei Key laboratory of Infectious Diseases Pathogenesis and Precise Diagnosis and Treatment, Baoding Hospital of Beijing Children’s Hospital, Capital Medical University, Baoding, Hebei Province, People's Republic of China; 2 Department of Pediatrics, Capital Medical University, Affiliated Beijing Friendship Hospital 26455, Beijing, People's Republic of China; 3 Beijing Key Laboratory of Pediatric Respiratory Infection Diseases, Key Laboratory of Major Diseases in Children, Ministry of Education, National Clinical Research Center for Respiratory Diseases, Laboratory of Respiratory Diseases, National Center for Children’s Health, Beijing Pediatric Research Institute, Beijing Children’s Hospital, Capital Medical University, Beijing, People's Republic of China; Tainan Hospital Ministry of Health and Welfare, Tainan, Taiwan

**Keywords:** respiratory pathogens, children, community-acquired pneumonia, non-pharmaceutical interventions lifting, Epidemiology

## Abstract

An observational, single-center study was conducted among hospitalized children with community-acquired pneumonia in the Baoding area. The objective was to compare the respiratory pathogen profiles and epidemiological patterns during and after the lifting of non-pharmaceutical interventions (NPIs, including mask-wearing, social distancing, lockdowns, etc.). A total of 9,362 hospitalized children diagnosed with CAP in Baoding from January 2022 to December 2023 were included. Both viral and bacterial pathogens were detected by multiplex PCR. The NPIs were lifted in Baoding from 2 December 2022. A comparative analysis was conducted on demographic data, epidemiological data, pathogen detection rates, and co-infection patterns that were compared between the full year 2022 and 2023. The detection rate of at least one pathogen significantly increased from 74.2% (2,925/3,940) in 2022 to 86.5% (4,690/5,422) in 2023 (*P* < 0.001). *Mycoplasma pneumoniae*, *Streptococcus pneumoniae*, human rhinovirus, respiratory syncytial virus (RSV), and *Haemophilus influenzae* were the predominant pathogens. Notably, co-infection rates rose from 25.1% (988/3,940) in 2022 to 45.1% (2,445/5,422) in 2023 (*P* < 0.001), particularly viral-bacterial co-infections. The positive detection rates for many pathogens among severe pneumonia cases increased. Additionally, an age shift in infection patterns was observed, with older children showing a higher prevalence of RSV. Following the lifting of NPIs, an increase in the detection rates of pathogens was observed, as were fluctuations in the dynamics of viral and bacterial pathogens, underscoring the urgent need to adapt clinical management and public health policies to address emerging infection trends.

**IMPORTANCE:**

Community-acquired pneumonia (CAP) remains the leading infectious cause of death in children worldwide. Understanding the pathogens responsible for CAP is essential for effective diagnosis and treatment. This study examines the changes in respiratory pathogens and epidemiological patterns in children with CAP in Baoding, China, before and after the lifting of non-pharmaceutical interventions (NPIs). Data from 9,362 children diagnosed with CAP from January 2022 to December 2023 were analyzed. The detection rate of at least one pathogen increased significantly from 74.2% in 2022 to 86.5% in 2023. Notably, co-infection rates rose from 25.1% to 45.1%, with viral-bacterial co-infections being more common. This research underscores the urgent need to adapt clinical management and public health policies to address the changing infection trends, highlighting their importance and innovation in understanding the impact of NPIs on pediatric respiratory infections.

## INTRODUCTION

Pneumonia remains the leading infectious cause of death in children worldwide, with over 725,000 deaths annually in children under 5, including 190,000 newborns (1). In China, an estimated 2 million children under 5 are diagnosed with pneumonia each year (2). Within this context, community-acquired pneumonia (CAP) poses a significant risk, contributing to high hospitalization rates, mortality, and economic burden within the pediatric population.

Understanding the pathogens responsible for CAP is essential for effective diagnosis and treatment. Pathogen profiles guide healthcare providers in selecting appropriate antimicrobial agents, reducing the risks of antibiotic overuse. The spectrum of pathogens varies by country, region, age group, season, and disease severity (3, 4). The COVID-19 pandemic has further complicated CAP management (5), with the decline in respiratory infections during strict non-pharmaceutical interventions (NPIs, including travel restrictions, social distancing, wearing masks, enhanced hygiene practices, temporarily school closures, workplace adjustments, public gathering restrictions, health screening and surveillance, etc.) followed by a resurgence of infections, especially in children, after the lifting of measures in December 2022 (6, 7).

Prior to 2022, studies identified a wide range of respiratory pathogens linked to pediatric pneumonia, with notable regional and seasonal variations (3, 8, 4). The preventive and control measures implemented during the COVID-19 pandemic had a significant impact on the spread of respiratory pathogens, affecting changes in their seasonality and epidemiological trends. For instance, respiratory syncytial virus (RSV) and influenza virus (IFV) infections exhibited a discernible seasonal peak prior to the pandemic, predominantly during the autumn and winter months. However, during the pandemic, there was a significant decline in the number of cases of RSV and IFV infections, with the seasonal peaks of infections being suppressed (9, 10, 11). Furthermore, IFV-B became the dominant pathogen during the 2021–2022 influenza season (10). Since the lifting of NPIs, research has focused on the rise of *Mycoplasma pneumoniae* infections (12, 13, 14), yet comprehensive data on CAP’s etiological and epidemiological features in children in China remain scarce post-NPI.

In response, we conducted a retrospective study on hospitalized children in Baoding to examine the pathogens associated with CAP during and after NPIs. The primary objective of this study was to elucidate the epidemiological characteristics and trends of pathogens associated with CAP in children after NPIs. Our results can be very useful for the improvement of empirical antibiotic treatment strategies for hospitalized children with CAP in this region.

## MATERIALS AND METHODS

### Patient enrollment and specimen collection

The study was conducted at Baoding Hospital of Beijing Children’s Hospital, Capital Medical University, a regional medical center serving Baoding and the surrounding areas. Patients aged 17 or younger, diagnosed with CAP and requiring hospitalization, were enrolled from January 2022 to December 2023. CAP diagnosis was based on clinical symptoms and radiographic findings (X-ray or computed tomography) consistent with guidelines for pediatric CAP (15).

Exclusion criteria included recent hospitalization (within 15 days), the administration of antibiotic treatment for a duration exceeding 3 days, an alternate respiratory diagnosis, tracheostomy tube, recent organ or stem-cell transplants, and conditions such as asthma, bronchiolitis obliterans, cystic fibrosis, or severe immunodeficiency.

Demographic and clinical data were collected, and specimens for pathogen detection (sputum, endotracheal aspirates, or bronchoalveolar lavage fluid [BALF]) were obtained and tested within 24 hours of hospitalization or stored at −80°C for later analysis. Enrolled patients were classified as severe CAP (SCAP) or non-severe CAP (non-SCAP) based on illness severity. Severe pneumonia was defined as a child with CAP exhibiting at least one of the following characteristics according to the Chinese guidelines (15): (i) poor general condition (a pale or gray complexion and a poor response to the surrounding environment); (ii) impaired consciousness; (iii) cyanosis; increased respiratory rate, ≥70 breaths/min in infants, ≥50 breaths/min in children >1-year old; assisted respiration, including groaning, nasal fanning, and triple concavity sign; intermittent apnea; and oxygen saturation <92%; (iv) ultra-high fever (41°C or higher), with persistent high fever (39°C or higher) for >5 days; (v) signs of dehydration or refusal to eat; (vi) ≥2/3 of one side of the lungs infiltrated, multilobar pulmonary infiltrates, pleural effusion, pneumothorax, pulmonary atelectasis, pulmonary necrosis, or pulmonary abscess; (vii) extrapulmonary complications.

### Laboratory procedures

Targeted viral pathogens included RSV, human rhinovirus (HRV), human bocavirus (HBoV), human metapneumovirus (HMPV), human adenovirus (HAdV), human parainfluenza virus (HPIV), IFV (IFV-A and IFV-B), and human coronavirus (HCoV). Bacterial pathogens tested included *Streptococcus pneumoniae*, *Acinetobacter baumannii*, *Staphylococcus aureus*, *Enterobacter cloacae*, *Pseudomonas aeruginosa*, methicillin-resistant *Staphylococcus aureus* (MRSA), *Klebsiella pneumoniae*, *Haemophilus influenzae*, *Escherichia coli*, *Stenotrophomonas maltophilia*, *Chlamydia pneumoniae*, and *M. pneumoniae*. Co-detection was defined as the presence of two or more pathogens in any combination.

The Respiratory Pathogen Multiplex Kit (Health Gene Technologies, Ningbo, China) was used to detect respiratory viral pathogens according to the recommended protocol. In brief, the nucleic acid was extracted from clinical specimens, and the extracted nucleic acids were subjected to reverse transcription PCR (RT-PCR). The products were then separated by capillary electrophoresis on a 3500Dx Genetic Analyzer (Applied Biosystems, USA). The peak height of the target site in the amplification product was found to exceed that of the capillary standard in the lane, and the site was consequently designated as positive. Multiplex fluorescent PCR assays were performed to detect bacterial pathogens using the Respiratory Pathogens Nucleic Acid Test Kit in accordance with the manufacturer’s instructions (Sansure Biotech, Changsha, China). The quality control measures implemented included the incorporation of positive and negative samples into the nuclear acid extraction process and each PCR run, thus ensuring the reliability of the experimental results.

### Data management and statistical analysis

Data on demographics, clinical presentation, lab results, and radiographic findings were collected from medical records and entered into a standardized database by trained clinicians. Five age groups were established: under 28 days, 29 days to 1 year, 2–4 years, 5–9 years, and 10–17 years. Detection rates were calculated as the percentage of children with specific pathogens detected by PCR. Descriptive statistics were used for categorical variables, with medians expressed as numbers (%). The NPIs were lifted on 2 December 2022 in Baoding. Therefore, all comparisons were made between the full years of 2022 and 2023. Comparative analyses of pathogen detection rates by sex, age, severity, and season were conducted using the *χ*^2^ test or Fisher’s exact test. Each variable was considered independently. Consequently, no adjustment (e.g., Bonferroni or false discovery rate) was implemented. Statistical analyses were performed using SPSS 25.0, with a *P*-value < 0.05 considered significant.

## RESULTS

### Demographic characteristics of patients

**Fig 1:**
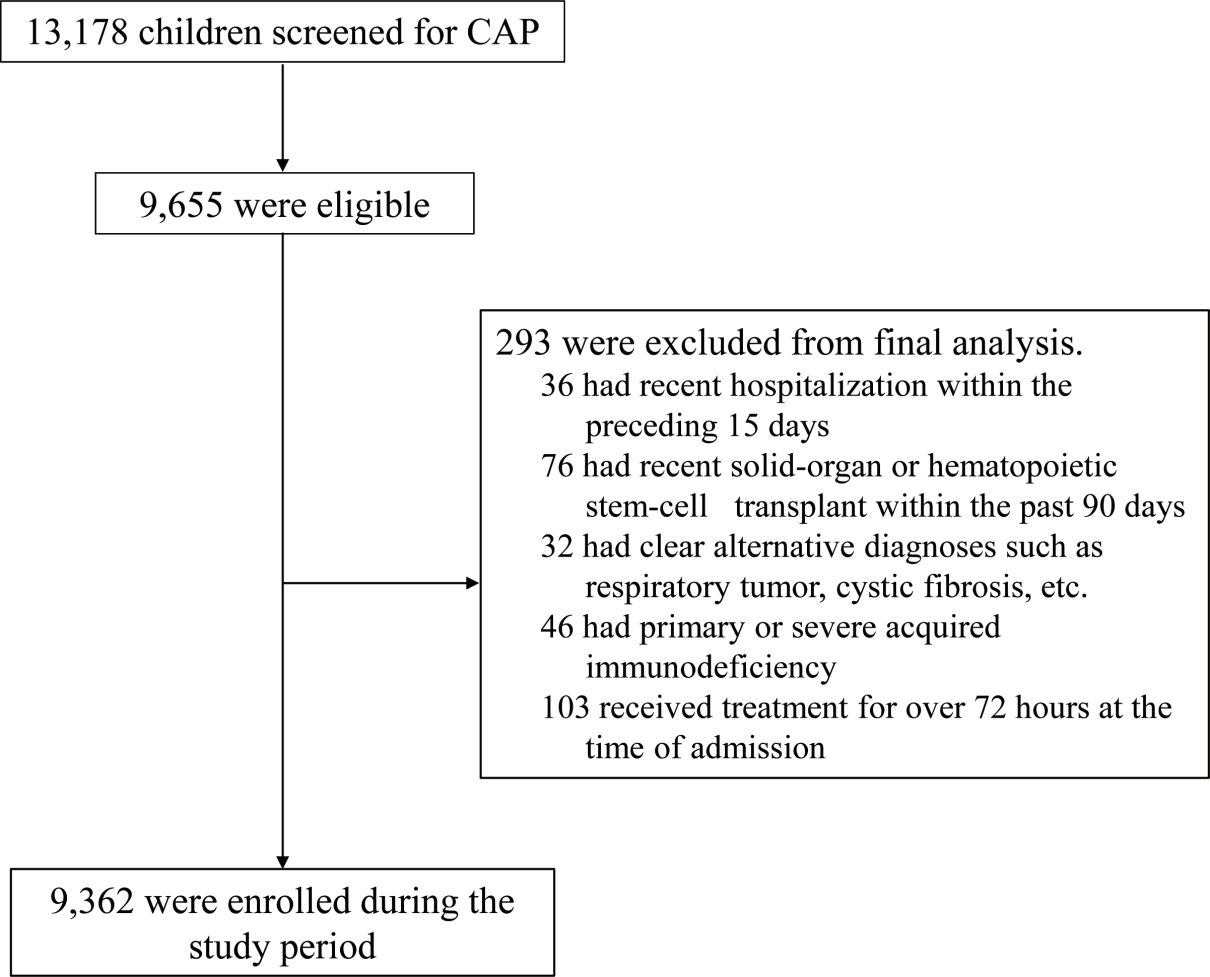
Children with community-acquired pneumonia enrollment flowchart.

From January 2022 to December 2023, 13,178 hospitalized children were screened, of whom 9,655 (73.3%) were initially diagnosed with CAP. After applying exclusion criteria, 9,362 participants were enrolled (Fig. 1).

Among the enrolled participants, 56.2% (5,263/9,362) were male, with a median age of 3 years (interquartile range [IQR] 1–6). The majority (90.6%) were aged 29 days to 9 years, with the highest proportion in the 5–9 year group (32.0%). Hospitalizations peaked in the fourth quarter of the year. SCAP was diagnosed in 14.1% (1,317/9,362) of cases.

Overall, 81.3% (7,615/9,362) of patients had at least one positive pathogen detected. Of these, 4,182 had single-pathogen infections, while 3,433 had co-infections (Table 1). In 2023, CAP cases significantly increased compared to 2022 (5,422 vs 3,940). While gender distribution remained unchanged, differences were observed in age groups, seasonal trends, and pathogen detection rates. The proportion of children >5 years increased in 2023 (42.2%) compared to 2022 (36.8%). Pathogen detection was higher in 2023 (86.5% vs 74.2% in 2022), with single-pathogen infections declining (49.2% in 2022 to 41.4% in 2023) and co-infections rising (25.1% to 45.1%, *P* < 0.001).

**TABLE 1:** Demographic characteristics of hospitalized patients with CAP

Characteristics	Total (*n* = 9,362)	2022 (*n* = 3,940)	2023 (*n* = 5,422)	*P* value
Gender, *n* (%)				
Male	5,263 (56.2)	2,253 (57.2)	3,010 (55.5)	0.108
Female	4,099 (43.8)	1,687 (42.8)	2,412 (44.5)	
Age group, *n* (%)				
≤28 days	142 (1.5)	68 (1.7)	74 (1.4)	0.158
29 days to 1 year	2,799 (29.9)	1,280 (32.5)	1,519 (28.0)	<0.001
2–4 years	2,687 (28.7)	1,144 (29.0)	1,543 (28.4)	0.542
5–9 years	2,994 (32.0)	1,184 (30.1)	1,810 (33.4)	<0.001
10–17 years	740 (7.9)	264 (6.7)	476 (8.8)	<0.001
Severity of CAP, *n* (%)				
SCAP	1,317 (14.1)	624 (15.8)	693 (12.8)	<0.001
Non-SCAP	8,045 (85.9)	3,316 (84.2)	4,729 (87.2)	
Quarter of year, *n* (%)				
Q1 (Jan–Mar)	1,405 (15.0)	1,055 (26.8)	350 (6.4)	<0.001
Q2 (Apr–Jun)	1,759 (18.8)	521 (13.2)	1,238 (22.8)	<0.001
Q3 (Jul–Sep)	2,323 (24.8)	1,073 (27.2)	1,250 (23.1)	<0.001
Q4 (Oct–Dec)	3,875 (41.4)	1,291 (32.8)	2,584 (47.7)	<0.001
Detection of pathogens, *n* (%)				
Any pathogen	7,615 (81.3)	2,925 (74.2)	4,690 (86.5)	<0.001
Any virus	4,730 (50.5)	1,540 (39.1)	3,190 (58.8)	<0.001
Any bacterium	5,605 (59.9)	2,151 (54.6)	3,454 (63.7)	<0.001
Single infection	4,182 (44.7)	1,937 (49.2)	2,245 (41.4)	<0.001
Co-detection	3,433 (36.7)	988 (25.1)	2,445 (45.1)	<0.001

### Pediatric CAP spectrum analysis

The detection rates of pathogens in 2022 and 2023 are shown in Table 2. In 2022, the most common pathogens were *M. pneumoniae*, *S. pneumoniae*, HRV, RSV, HMPV, and IFV. Following the lifting of NPIs, CAP cases rose significantly. Notable increases were observed in HAdV (1655.0%), *H. influenzae* (244.6%), HCoV (208.5%), RSV (178.9%), and HBoV (177.0%) in 2023 compared to 2022.

**TABLE 2:** Annual cases and change in proportion of different pathogens in pediatric CAP patients

Pathogens	2022 (*N* = 3,940) *n*%	2023 (*N* = 5,422) *n*%	Change %^*a*^	*P* value
Bacteria				
*M. pneumoniae*	1,034 (26.2)	1,663 (30.7)	60.8	<0.001
*S. pneumoniae*	933 (23.7)	1,426 (26.3)	52.8	0.004
*H. influenzae*	231 (5.9)	796 (14.7)	244.6	<0.001
*S. aureus*	84 (2.1)	84 (1.5)	0.0	0.036
*K. pneumoniae*	54 (1.4)	69 (1.3)	27.8	0.681
*A. baumannii*	34 (0.9)	55 (1.0)	61.8	0.456
MRSA	32 (0.8)	28 (0.5)	−12.5	0.077
*E. cloacae*	24 (0.6)	24 (0.4)	0.0	0.266
*S. maltophilia*	9 (0.2)	3 (0.1)	−66.7	0.036
*E. coli*	8 (0.2)	9 (0.2)	12.5	0.807
*P. aeruginosa*	7 (0.2)	12 (0.2)	71.4	0.817
*Chlamydia pneumoniae*	3 (0.1)	4 (0.1)	33.3	1.000
Viruses				
HRV	399 (10.1)	918 (16.9)	130.1	<0.001
RSV	336 (8.5)	937 (17.3)	178.9	<0.001
HMPV	283 (7.2)	408 (7.5)	44.2	0.532
IFV	269 (6.8)	400 (7.4)	48.7	0.308
HPIV	169 (4.3)	398 (7.3)	135.5	<0.001
HBoV	100 (2.5)	277 (5.1)	177.0	<0.001
HCoV	47 (1.2)	145 (2.7)	208.5	<0.001
HAdV	20 (0.5)	351 (6.5)	1,655.0	<0.001

^*a*^ The change proportion of annual cases was calculated as: (Cases_2023_ – Cases_2022_)/Cases_2022_ × 100%.

**Fig 2:**
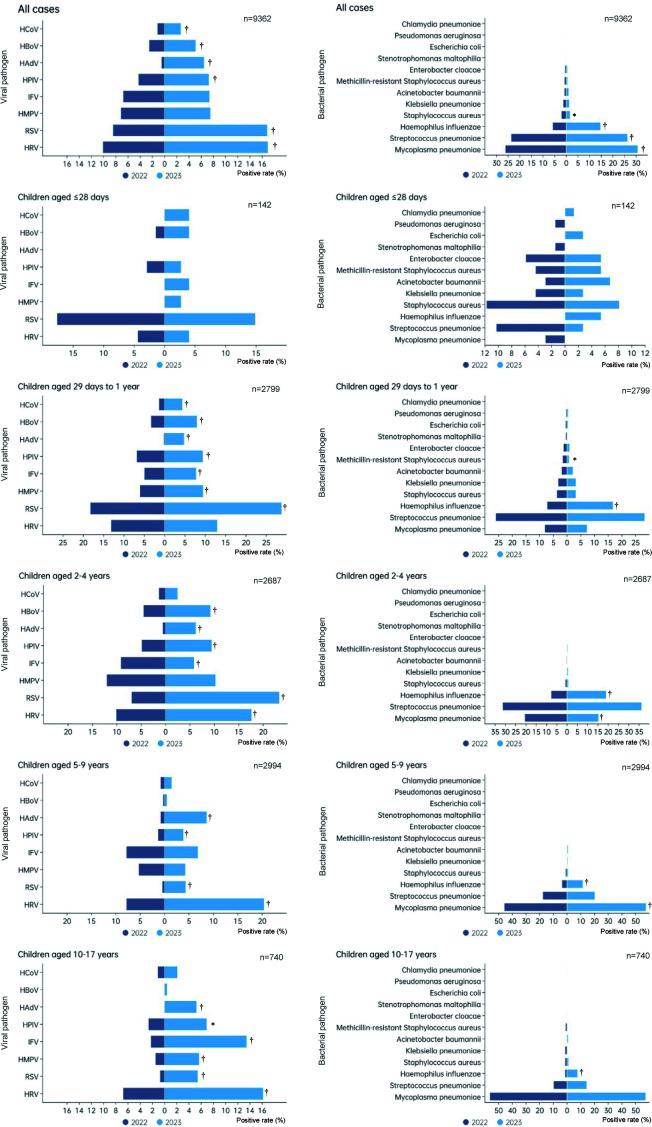
Comparison of the positive rates of 8 viral and 12 bacterial pathogens in hospitalized patients with CAP in 2022 and 2023. The positive rate of each pathogen among 3,940 hospitalized CAP patients in 2022 and 5,422 patients in 2023 was compared for different age groups. The length of the light blue bar indicates the positive rate in 2023, and the length of the dark blue bar indicates the positive rate of 2022. The positive rate was calculated by taking the positive number of each pathogen as the numerator and the number of CAP tested as the denominator. The significant difference in the positive rate (*χ*^2^ test or Fisher’s exact test) is indicated. *P < 0.05 and †*P* < 0.01.

Pathogen detection rates varied by age (Fig. 2). Viral detection increased significantly in children aged 29 days and older in 2023, with RSV, HAdV, and HPIV showing marked increases. HRV detection rates increased significantly in children older than 2 years, while HBoV saw notable increases in children aged 29 days to 4 years. IFV detection was highest in the 10–17 year group. Bacterial detection rates also varied, with *H. influenzae* increasing across all age groups and *M. pneumoniae* rising notably in children aged 2–9 years.

### Epidemiological features of different pathogens

**Fig 3:**
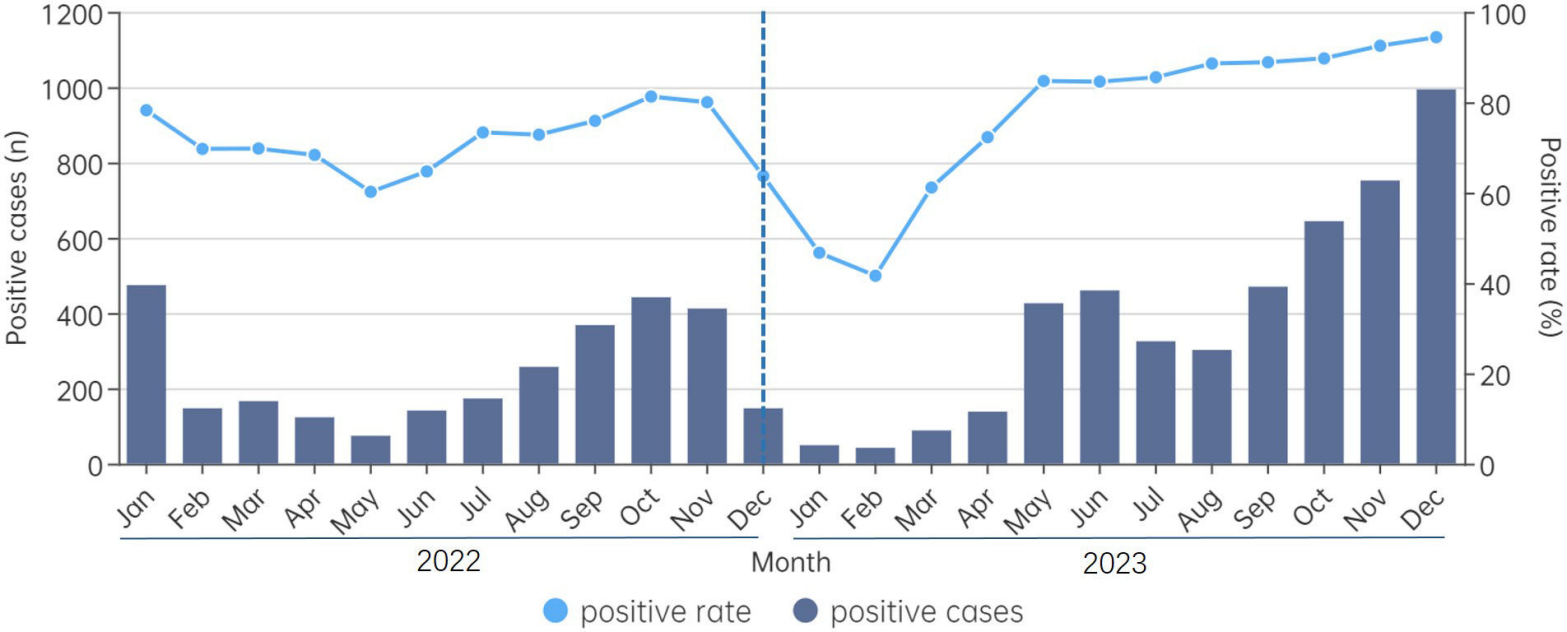
Cases and rates of pathogen-positive detection by month. The dotted line represented the positive rate of any pathogen, while the bar illustrated the number of positive cases observed in each month. The vertical line represents the time of the lifting of the NPI.

**Fig 4:**
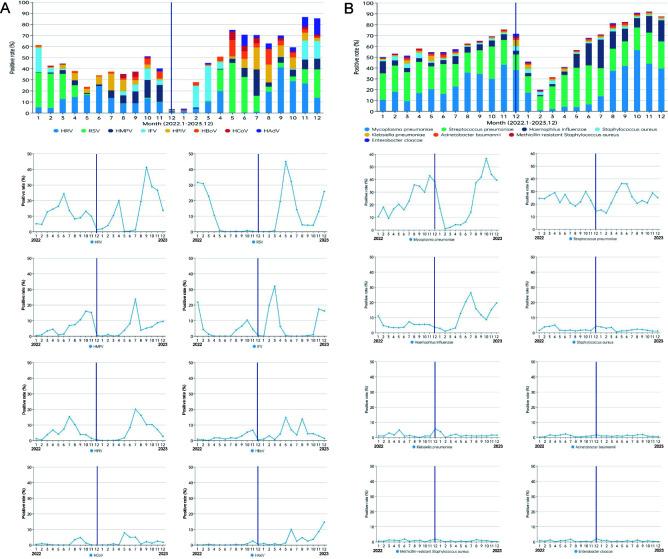
The detection rate of different pathogens in hospitalized children with CAP by month. (**A**) Viral pathogens. (**B**) Bacterial pathogens.

Epidemiological patterns of CAP-related pathogens were analyzed (Fig. 3). Following the lifting of NPIs in December 2022, viral pathogen detection dropped sharply but rebounded by early 2023. As shown in Fig. 4, IFV was the first to re-emerge, followed by HRV and RSV. Seasonal patterns changed: HRV peaked twice in 2023, RSV shifted from winter-spring to spring-summer, and IFV peaked in March rather than winter. Further analysis revealed that IFV-B was predominant (53.9%, 145/269) in 2022, while IFV-A became the dominant type in 2023 (97.0%, 388/400).

Bacterial pathogen detection decreased after NPIs were lifted, with *M. pneumoniae* showing a delayed peak from June 2023. *S. pneumoniae* exhibited steady fluctuations, while *H. influenzae* detection increased significantly in 2023.

### Co-infection patterns

**Fig 5:**
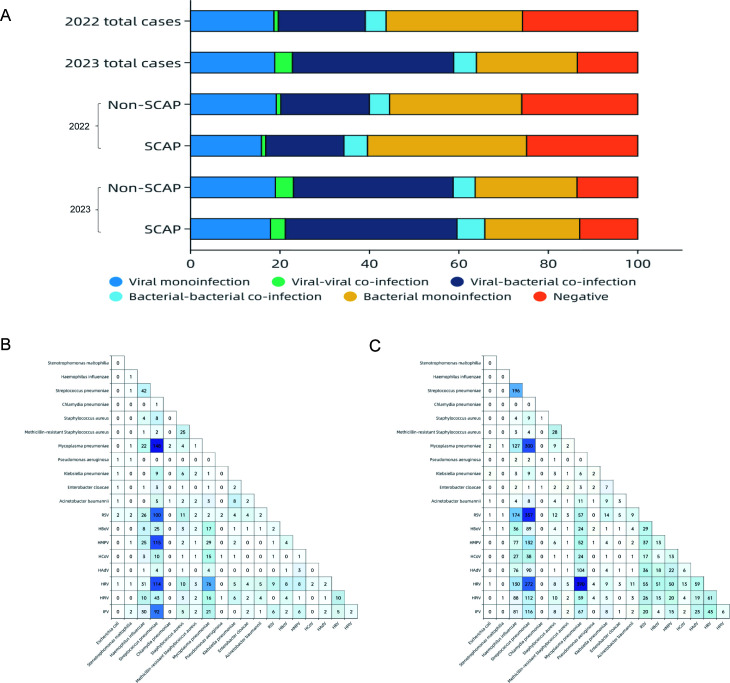
Prevalence of pathogens in mono-infection and co-infection in children with CAP during 2022 and 2023. (**A**) Positive proportion of viruses, viral-viral co-infections, viral-bacterial co-infections, bacterial-bacterial co-infections, and bacteria in different years. (**B** and **C**) Heatmap of the co-infection rate of respiratory pathogens in 2022 (**B**) and 2023 (**C**). The grid color represents the co-infection rate of respiratory pathogens among children with SCAP. A darker color indicates higher co-infection rates between the pair of pathogens.

Among the 9,362 enrolled children, 3,433 (36.7%) had co-infections: 2,720 (29.1%) with viral-bacterial, 254 (2.7%) with viral-viral, and 459 (4.9%) with bacterial-bacterial co-infections (Fig. 5A). Co-infection rates increased from 25.1% in 2022 to 45.1% in 2023, particularly viral-bacterial co-infections (19.4%–36.0%, *P* < 0.001). The largest increases were observed for HRV-*M. pneumoniae*, followed by RSV-*S. pneumoniae*, HRV-*S. pneumoniae*, *S. pneumoniae-H. influenzae*, and *M. pneumoniae-S. pneumoniae* (Fig. 5B and C). However, the co-infections were not associated with greater clinical severity (Fig. 5A).

### Severe CAP-associated pathogens

**Fig 6:**
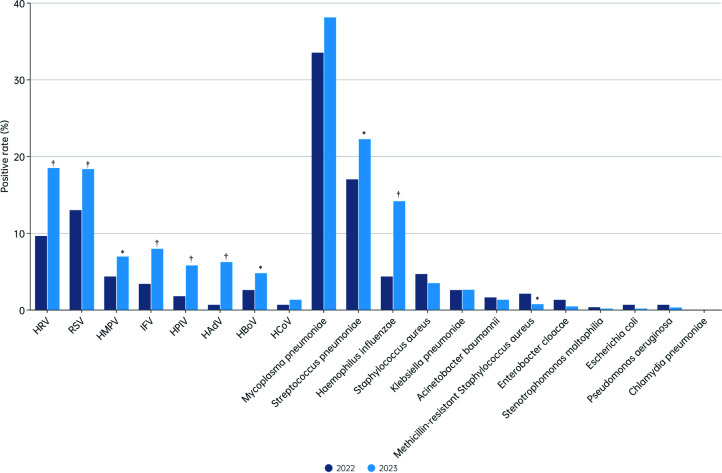
Comparison of positive rates of different pathogens in children with severe CAP between 2022 and 2023. The number of severe cases in 2022 and 2023 was 624 and 693, respectively. *P < 0.05 and †*P* < 0.01.

The detection rates for severe CAP patients in 2022 and 2023 are shown in Fig. 6. All viral pathogens, except HCoV, showed significantly increased detection rates in 2023, with the largest increases for HAdV (from 0.6% to 6.2%), HPIV (from 1.8% to 5.8%), IFV (from 3.4% to 7.9%), HRV (from 9.6% to 18.5%), and RSV (from 13.0% to 18.3%). Among bacterial pathogens, *H. influenzae* and *S. pneumoniae* showed the largest increases (from 4.3% to 14.1% and from 17.0% to 22.2%, respectively). The increase in *M. pneumoniae* detection was not statistically significant (33.5% in 2022 vs 38.1% in 2023, *P* = 0.08).

## DISCUSSION

This study provides a comprehensive analysis of respiratory infections in the Baoding area during and after the implementation of NPIs, focusing on pediatric patients hospitalized with CAP at a large medical center. We observed significant shifts in the pathogen profiles and epidemiological patterns of respiratory infections across various age groups, both during and after the NPIs.

Geographical and seasonal variations are well-known in respiratory pathogen prevalence, with certain pathogens circulating year-round. The implementation of NPIs effectively reduced the transmission of respiratory infections (7), but their lifting in December 2022 resulted in a surge in COVID-19 cases, which subsequently altered the pathogen spectrum and epidemic patterns. Our findings demonstrate a rise in pathogen detection rates from 74.2% in 2022 to 86.5% in 2023, consistent with trends reported by other studies (6, 8), where pathogen detection decreased during NPIs and increased after their lifting.

In 2022, the predominant pathogens among hospitalized children with CAP in Baoding were *M. pneumoniae*, *S. pneumoniae*, HRV, RSV, and HMPV. In 2023, *M. pneumoniae*, *S. pneumoniae*, HRV, and RSV still remained dominant, while *H. influenzae* also became predominant (Table 2). A pre-COVID-19 national survey (2009–2019) reported that the leading viruses were RSV, HRV, and HPIV, with *S. pneumoniae*, *H. influenzae*, and *M. pneumoniae* as the most common bacteria (16). The pandemic had a notable impact on pediatric pneumonia, particularly with a rise in *M. pneumoniae* cases and a decrease in RSV ranking. Previous data from the Beijing area showed average positive rates for *M. pneumoniae* DNA testing of 24.4% from 2006 to 2015 and 28.1% from 2016 to 2019 (17, 18). Even after NPIs, the positive rate for *M. pneumoniae* IgM testing remained high at 24.27% among children with acute respiratory infections (19). This aligns with our findings: the detection rate of *M. pneumoniae* rose from 26.2% in 2022 to 30.7% in 2023 (Table 2), raising concerns about immunity debt—where NPIs prevented natural pathogen circulation, limiting population immunity (20). The immunity debt likely contributed to the surge in respiratory infections, especially viral pathogens in children. Because immunity generated by infection with viral pathogens is relatively short-lived and without seasonal outbreaks, immunity decreases, and susceptibility to future, and potentially more severe, infections increases (21, 22, 23). This phenomenon is corroborated by the substantial surge in the number of cases of viral infections observed in 2023 in this study.

Following the lifting of NPIs in December 2022, we observed an initial decline in pathogen detection rates, particularly for viral pathogens, which dropped sharply in December, while bacterial pathogens reached their lowest point in February 2023. This unusual decline may be explained by viral interference, where the presence of one virus inhibits the replication of others (24). This interaction could involve immune responses (25, 26), competition for host cell resources (27), and downregulation of cellular receptors (28), which likely contributed to the rapid decline in viral detections after the surge in COVID-19 cases.

After the NPIs were lifted, the epidemic patterns of several pathogens shifted. IFV was the first to rebound, with a peak in March 2023 and a smaller peak in November. This pattern deviates from historical trends, where IFV typically peaks in winter. However, similar anomalous increases during off-peak seasons were observed in a multi-center adult study in China (29). Our data showed that in 2022, IFV-B was predominant, but in 2023, IFV-A became the dominant strain. According to the World Health Organization FluNet surveillance, IFV-B peaked in January 2022, with IFV-A peaking in July 2022 and March 2023, and rose again in October 2023 (30). Historically, IFV has posed a significant threat, primarily due to pandemics caused by IFV-A (31); however, IFV-B has not yet caused a pandemic. This shift may reflect the pandemic’s disruption of typical viral circulation patterns, with IFV-B re-emerging after IFV-A was suppressed during the COVID-19 period (10).

In our study, HRV was consistently present throughout 2022 and exhibited two peaks in 2023, consistent with previous reports indicating its persistence in regions like California and South Korea during the pandemic (32, 33). A nationwide analysis of pediatric data in Finland revealed that HRV detection decreased initially with early lockdowns but returned to typical levels as restrictions were lifted (34). The inherent characteristics of HRV, including its non-enveloped nature, surface stability, and genomic diversity, may allow for rapid increases in infections once NPIs are relaxed.

RSV, which traditionally peaks in winter (35), saw a significant increase in cases starting in March 2023, reaching a high point in May and rising again in November. This shift in RSV’s seasonal prevalence was also observed in Yunnan, with altered clinical features and increased severity (36). In 2023, the age distribution of RSV patients shifted toward older children, a trend seen in Yunnan and Australia (36, 37). Besides the previously mentioned immunity debt phenomenon, a decrease in antibody concentrations among older children may explain the pronounced surge in RSV cases in this age group (38).

A remarkable rise in HAdV cases was also observed in 2023, with a staggering increase of 1,655.0% (from 20 in 2022 to 351 in 2023). The detection rate of HAdV rose from 0.5% (20/3940) in 2022 to 6.5% (351/5422) in 2023, with peaks in June and December. A previous study of 1,778 hospitalized children in Guangzhou reported an infection rate of 2.5% from 2013 to 2017 and 6.0% from 2018 to 2019 (39). The prevalence of HAdV appears to have significantly declined during the NPIs period, only to return to elevated levels shortly after their lifting, aligning with epidemiological patterns observed by Xu et al. (7).

Bacterial pathogens exhibited a smaller decline post-NPI, with *M. pneumoniae* and *H. influenzae* showing the most pronounced changes. *M. pneumoniae* continues to attract attention due to its increasing detection rates across China. Prior studies noted that *H. influenzae* exhibited seasonal distribution patterns, with increases during winter months (40). In our study, *H. influenzae* experienced a notable resurgence in 2023, with an increase of 244.6% (from 231 in 2022 to 796 in 2023), suggesting a resurgence of this common pneumonia pathogen. In comparison with viral pathogens such as IFV, *H. influenzae* and *M. pneumoniae* demonstrated a delayed peak after COVID-19 pandemic restrictions. The potential reasons for *H. influenzae* might be the viral-bacterial interactions as observed for *S. pneumoniae*, when the temporal suppression of RSV and IFV was associated with a decline in pneumococcal disease in young children (41). However, *M. pneumoniae* has been observed to exhibit a longer delay following the re-emergence of respiratory viruses. One hypothesis for this delay is that herd immunity might be responsible. Previous data have indicated an interval of 3–7 years between *M. pneumoniae* epidemics. The most recent epidemic period of *M. pneumoniae* was in 2019. The transient herd immunity might have led to the delayed re-emergence. Furthermore, the atypical characteristics of *M. pneumoniae*, including the slow generation time (6 hours), the long incubation period (1–3 weeks), and the relatively low transmission rate, could also be factors leading to a longer time interval required for the re-establishment of *M. pneumoniae* infection within a population (42).

Our study also identified a high incidence of co-infections, with a significant increase from 25.1% in 2022 to 45.1% in 2023. The etiology of this condition is multifactorial. Initially, following the lifting of NPIs, the majority of children were infected with COVID-19. This may have resulted in an immune dysregulation, which could have contributed to subsequent secondary infections (43). Second, as previously stated, the reduction in pathogen exposure during the NPI period resulted in an immune debt. Third, the synergistic effect between pathogens must be considered. In the present study, viral-bacterial co-infections were the most prevalent, while co-infections between viruses were relatively rare, a finding that was consistent with previous reports (3, 4). The mechanisms underlying viral-bacterial co-infection may include viral infections causing airway damage, promoting bacterial adherence, and disrupting host immune regulation (44, 45). Conversely, it has been demonstrated that bacterial infections may predispose individuals to viral infections by facilitating viral propagation within the respiratory system (46). The present study revealed that the most prevalent combinations were HRV-*M. pneumoniae* and RSV-*S. pneumoniae*. A study conducted in Soochow found a 7.6% co-infection rate of HRV and *M. pneumoniae* in children with acute respiratory infections in 2023 (7). Another review highlighted significant biological and clinical interactions between pneumococcus and RSV in the pathogenesis of childhood respiratory infections (47). These co-infections may contribute to the increased burden of respiratory infections following the lifting of NPIs.

Interestingly, the proportion of children with SCAP decreased in 2023 (12.8%) compared to 2022 (15.8%), yet the positive detection rates of many viruses and some bacteria in SCAP cases increased. This trend could be attributed to the higher overall pathogen detection rate in 2023. Previous studies have linked SCAP to infections with HAdV, HRV, RSV, *H. influenzae*, and *Klebsiella pneumoniae* (4, 48, 49), and similar associations were noted in our data. Notably, despite the considerable increase in *M. pneumoniae* infections in 2023, its positive rate among severe cases did not show a significant rise.

This study has several limitations. First, during the long study period, the enrolled patients typically submitted a single specimen for detection, given the expense associated with respiratory multipathogen testing. It is acknowledged that the diagnostic yield may vary according to the type of specimen. Nonetheless, the specific specimen type for each patient was not documented, which has the potential to result in under- or overestimation of pathogen-specific detection rates. Second, in the present study, molecular assays were used exclusively to detect bacterial pathogens, which may result in a higher positive rate than conventional culture methods. In addition, our findings do not establish causal relationships, particularly with regard to bacterial pathogens, since the detection of pathogens in BALF and sputum does not definitively distinguish between colonization and invasive infection. Third, the commercial kit was employed to facilitate the detection of 12 bacteria and 8 viruses. However, it should be noted that the panel did not include all respiratory pathogens, which may result in an underestimation of the detection rates and co-infection rate. Furthermore, the utilization of disparate detection methodologies gives rise to distinct thresholds, which in turn can exert an influence on positivity rates.

### Conclusion

This study highlights significant changes in respiratory pathogen profiles and epidemiological patterns among hospitalized children with CAP in Baoding during and after the NPIs. The lifting of NPIs was associated with increased pathogen detection rates, with *M. pneumoniae* emerging as a dominant pathogen. The dynamics of viral and bacterial pathogens, including RSV, HRV, and *H. influenzae*, demonstrated considerable fluctuations post-NPI. The rise in co-infections, particularly viral-bacterial combinations, underscores the complex interplay between pathogens and suggests a shift in susceptibility due to immunity debt. These findings had useful implications for clinical management. The empirical treatment should consider the increase of *M. pneumoniae* and *H. influenza*. It is imperative to consider the vaccination of *S. pneumoniae* and IFV in children. Furthermore, the utilization of comprehensive pathogen panels is recommended for diagnostic purposes in clinical settings. These findings also underscore the necessity for sustained surveillance and the implementation of adaptive public health strategies to address the resurgence of respiratory infections in children.
